# Upregulated FASN-mediated lipogenesis in senescent macrophages contributes to liver fibrosis progression

**DOI:** 10.3389/fimmu.2026.1863063

**Published:** 2026-07-14

**Authors:** Hongliang Dong, Chuanfang Shu, Ran Liu, Mingpei Zhao, Kaiyue Zhang, Ziqun Qu, Lili Wang, Jing Fan, Wei Ye

**Affiliations:** 1 Department of Infectious Disease and Liver Disease, The Second Hospital of Nanjing, Affiliated to Nanjing University of Chinese Medicine, Nanjing, Jiangsu, China; 2 Nanjing Key Laboratory of Hepatology Medicine, The Second Hospital of Nanjing, Affiliated to Nanjing University of Chinese Medicine, Nanjing, Jiangsu, China

**Keywords:** cellular senescence, fatty acid metabolism, hepatic stellate cells, liver fibrosis, macrophages

## Abstract

**Background:**

Liver fibrosis represents a common advanced pathological stage of various chronic liver diseases. Macrophages serve as key regulators of innate immunity and play important roles in the development of liver fibrosis. Cellular senescence is an irreversible cell cycle arrest state. However, the mechanisms underlying the role of senescent macrophages in liver fibrosis remain incompletely understood.

**Methods:**

Liver fibrosis was induced in young (8-week) and middle-aged (12-month) mice by intraperitoneal injection of 25% carbon tetrachloride (CCl_4_). Hepatic function and the immune microenvironment were evaluated using histopathology, serum biochemistry, Western blotting, RT-qPCR, immunohistochemistry, and immunofluorescence. A senescent macrophage model was established in RAW264.7 cells by exposure to H_2_O_2_. Conditioned medium from senescent macrophages was transferred to LX-2 cells to assess hepatic stellate cell (HSC) activation. Transcriptome sequencing of senescent RAW264.7 cells was performed to identify underlying mechanisms. FASN protein stability was examined using cycloheximide (CHX) chase assays, and degradation pathways were preliminarily explored by combined treatment with MG132 and chloroquine (CQ).

**Results:**

*In vivo*, middle-aged mice treated with CCl_4_ exhibited more severe hepatic collagen deposition, fibrosis, and senescence than young mice. *In vitro*, senescent macrophages showed upregulated expression of SASP components, including pro-inflammatory cytokines and chemokines, and DNA damage markers, and their conditioned medium promoted LX-2 cell activation. Transcriptome sequencing of senescent RAW264.7 cells revealed a bidirectional fatty acid metabolic reprogramming characterized by downregulation of genes involved in fatty acid β-oxidation and upregulation of genes involved in fatty acid synthesis. Furthermore, FASN protein was found to undergo dual degradation via both the ubiquitin-proteasome and autophagy pathways. Moreover, pharmacological inhibition of FASN attenuated the DNA damage response in senescent macrophages.

**Conclusion:**

Macrophages in middle-aged fibrotic livers exhibit cellular senescence. FASN-mediated enhancement of fatty acid synthesis leads to lipid metabolic disorder and accumulation in senescent macrophages. These senescent macrophages promote HSC activation and drive the progression of liver fibrosis.

## 1 Introduction

Liver fibrosis is a pathological condition resulting from chronic liver damage and inflammation. Its causes are multifaceted, including viral infections, alcohol abuse, and drug toxicity (1). As a major global health concern, this disorder is characterized by excessive deposition of extracellular matrix (ECM) and structural disorganization in the liver (2, 3), ultimately progressing to life-threatening stages such as cirrhosis and hepatocellular carcinoma. Globally, it claims approximately 2 million lives annually (4). Therefore, it is of great significance to study the pathological mechanism and treatment of liver fibrosis in clinical practice and medical research.

Cellular senescence, an irreversible state of cell cycle arrest, can be triggered by multiple factors including aging, DNA damage, oncogene activation, and oxidative stress. The molecular mechanisms underlying senescence involve the p16, p21 and p53 tumor suppressor pathways, telomere shortening, and various complex cellular changes (5). Additionally, cellular senescence is recognized as a potent tumor-suppressive barrier that limits the proliferation of damaged cells (6). Senescent cells can influence neighboring cells by secreting a senescence-associated secretory phenotype (SASP) comprising pro-inflammatory mediators (7). The conventional understanding holds that immune cells target and clear senescent cells. However, recent studies reveal that immune cells themselves can undergo senescence, promoting the spread of paracrine senescence and thus contributing to systemic aging and age-associated pathologies (8).

Macrophages have been identified as key regulators of hepatic inflammation and critical factors in the progression or regression of liver fibrosis (9). Activated macrophages release cytokines that damage hepatocytes, increase inflammatory cell infiltration, and activate hepatic stellate cells (HSCs) (10).The diverse, complex, and crucial pathways through which macrophages influence HSCs play a pivotal role in the development of hepatic fibrosis (11).

Previous research demonstrated causally that an aged immune system is sufficient to induce systemic aging and tissue senescence, highlighting immune aging as a central mechanism of organismal decline (8). Macrophages are essential for innate immunity and tissue homeostasis, yet they are susceptible to cellular senescence induced by aging, metabolic stress, and chronic inflammation (8, 12). Senescent macrophages display a SASP, defective phagocytosis, and metabolic reprogramming, thereby driving inflammation and accelerating age-related disorders such as atherosclerosis, neurodegeneration, and cancer. Consequently, strategies aimed at eliminating senescent macrophages or modulating the SASP are being actively explored to mitigate age-associated pathologies (12–14). Despite this progress, the influence and possible mechanism of senescent macrophages in the occurrence and development of hepatic fibrosis have been less explored.

Here, we aimed to investigate the role of senescent macrophages in liver fibrosis and to elucidate the underlying mechanisms. We employed multiple *in vivo* approaches to determine their presence in fibrotic livers, assess their impact on hepatic stellate cell activation, and identify key regulatory pathways to provide a therapeutic rationale for targeting senescent macrophages in fibrosis.

## 2 Materials and methods

### 2.1 Animal

Male C57BL/6 mice (young: 8 weeks, approximately 20 g; middle-aged: 12 months, approximately 30 g) were obtained from Shanghai Model Organisms Center, Inc. (Shanghai, China). Animals were housed under specific pathogen-free conditions at 22–26 °C, 40–70% humidity, and a 12-hours light/dark cycle, with free access to standard chow and sterile water. Mice were randomly assigned to control and experimental subgroups within each age cohort. Liver fibrosis was induced by intraperitoneal injection of CCl_4_ (RHAWN, Shanghai, China) dissolved in corn oil (1:3 v/v; Aladdin, Shanghai, China) using an escalating dose regimen: 1.75 mL/kg (first dose), 3.50 mL/kg (2nd–9th doses), and 5.0 mL/kg (10th–24th doses), administered three times weekly over eight weeks (15). Control animals received an equivalent volume of corn oil alone. Twenty-four hours after the final injection, mice were anesthetized with isoflurane, and blood was collected from the retro-orbital plexus. Animals were subsequently euthanized by cervical dislocation, and liver and blood samples were harvested for biochemical, histological, and gene expression analyses. All procedures were approved by the Institutional Animal Care and Use Committee of Nanjing University of Chinese Medicine and conducted in accordance with institutional and national guidelines for the care and use of laboratory animals.

### 2.2 Human liver tissue samples

Of the 12 participants enrolled in this study at the Liver Cirrhosis Treatment Center of the Second Hospital of Nanjing from November 2023 to November 2025, 6 were diagnosed with hepatitis and 6 with liver fibrosis S3. Liver tissue specimens were obtained through percutaneous liver biopsy and embedded in paraffin (**Supplementary Table 3**). Patients with liver fibrosis S3 were diagnosed using histological, clinical and imaging criteria. With the approval of the Medical Ethics Committee of the Second Hospital of Nanjing, all participants provided written informed consent in accordance with the Helsinki Declaration (2013). All research procedures were conducted in accordance with relevant guidelines and regulations.

### 2.3 Histological evaluation

Fresh mouse liver samples were fixed in 4% paraformaldehyde (PFA) (Proteinbio, Nanjing, China) for 24 hours. The tissue was then dehydrated and embedded in paraffin, cut into 5 μm-thick sections, and stained with hematoxylin and eosin (H&E) for histological examination at room temperature. Fibrosis assessment was performed using Masson’s trichrome and Sirius red stains (Baso, Zhuhai, China) according to the manufacturer’s protocol. Finally, the sections were sealed with neutral resin and scanned and photographed with a light microscope for all the prepared sections.

### 2.4 Blood biochemical assessment

Mouse blood was collected and allowed to clot overnight at 4 °C. The sample was then centrifuged at 3000 rpm for 15 minutes at 4 °C to obtain serum, which was subsequently sent to Wuhan Servicebio Technology Co., Ltd. for analysis of liver function parameters, including alanine aminotransferase (ALT), aspartate aminotransferase (AST), alkaline phosphatase (ALP), total bilirubin (TBIL), direct bilirubin (DBIL), and gamma-glutamyl transferase (γ-GT).

### 2.5 Cell culture and processing

RAW264.7 macrophage cells (Zhongqiao Xinzhou, Shanghai, China) and LX-2 human hepatic stellate cells (Zhongqiao Xinzhou, Shanghai, China) were cultured in DMEM high-glucose medium supplemented with 10% fetal bovine serum (Absin, Shanghai, China) and 1% penicillin-streptomycin (Proteinbio, Nanjing, China) at 37 °C under 5% CO_2_. A cellular senescence model was established by inducing DNA damage in RAW264.7 cells through 200 μmol/L H_2_O_2_ (MACKLIN, Shanghai, China) (16). The optimal concentration was identified through CCK-8 assay (DOJINDO, Kumamoto-ken, Japan).

### 2.6 Cell co-culture

Senescent conditioned medium (CM) was prepared from RAW264.7 cells with the experimental group induced by 200 μmol/L H_2_O_2_ for 2 hours to establish senescence and the control group receiving PBS. After 24 hours, the supernatant was collected.

LX-2 cells were seeded at 2×10^5^ cells per well in a 6-well plate and cultured for 24 hours. The cells were then divided into five groups for 48 hours treatment: (1) LX-2, (2) LX-2 + H_2_O_2_, (3) LX-2 + CM from control RAW264.7, (4) LX-2 + CM from senescent RAW264.7.(5) LX-2 + 10 ng/mL TGF-β, Protein and RNA were then extracted for Western blotting and RT-qPCR analysis.

### 2.7 Western blot analysis

Total protein was extracted from mouse liver tissue and RAW264.7 cells using the Cell Protein Extract Kit (KeyGen BioTECH, Nanjing, China). Protein concentration was measured with the BCA Protein Assay Kit (ProteinBio, Nanjing, China).

Proteins were separated using a 8%, 10% or 12% SDS-PAGE gel and then transferred to a 0.22 μm PVDF membrane. The membrane was blocked for 2 hours with 5% skim milk dissolved in TBST and then incubated with the primary antibody overnight at 4 °C. The primary antibodies included TUBULIN, GAPDH, γH2AX, p53, phospho-p53, RB, phospho-RB, p19, p21, 53BP1, IL-1β, TNF-α, α-SMA, COL1A1, FASN, ACACA, CPT2, HDAC2, p38, PPAR δ and PPAR γ. The antibodies were from Abcam (Cambridge, UK) and Cell Signaling Technology (CST; Danvers, MA, USA). After three washes with TBST, the membrane was incubated with goat anti-rabbit IgG (H+L) (HRP) secondary antibody (ABclonal, Wuhan, China). ECL kit (ABclonal, Wuhan, China) was then used to evaluate the bands. The optical density of target protein bands were quantified using Image J 1.8 software (Rawak Software, Stuttgart, Germany).

### 2.8 Quantitative real-time PCR analysis

Total RNA was extracted from mouse liver tissue or RAW264.7 cells using the FastPure Cell/Tissue Total RNA Isolation Kit V2 (Vazyme, Nanjing, China), and the RNA concentration in each sample was measured. cDNA was synthesized from the RNA using HiScript III All-in-one RT SuperMix Perfect for qPCR kit (Vazyme, Nanjing, China). PCR amplification was performed using the ChamQ SYBR qPCR Master Mix (Vazyme, Nanjing, China) on a Cobasz480 (Roche, Basel, Switzerland) for 40 cycles. GAPDH, the housekeeping gene, was used for standardizing gene expression. Each experiment was repeated three times with three technical replicates per sample. The 2^-△△Ct^ method was employed to quantify gene expression levels, with *GAPDH* serving as the reference gene.

### 2.9 Immunohistochemistry

Paraffin-embedded sections were baked, deparaffinized, and rehydrated. Antigen retrieval was performed in sodium citrate buffer (pH 6.0). After blocking with 3% BSA, sections were incubated overnight at 4 °C with primary antibodies, followed by incubation with an HRP-conjugated secondary antibody at room temperature for 1 hour. Immunoreactivity was visualized using DAB substrate. Finally, the slides were sealed with neutral resin and observed under a light microscope (Axio Imager A2, Zeiss, Oberkochen, Germany).

### 2.10 Immunofluorescence

Paraffin-embedded tissue sections underwent dewaxing and dehydration, followed by antigen retrieval in Tris-EDTA buffer (pH 9.0) using a pressure cooker for 10 minutes. After cooling and PBS rinses, sections were blocked with 3% BSA for 1 hour at room temperature, then incubated with primary antibody at 4 °C overnight. After PBS washes, Alexa Fluor 488-conjugated secondary antibody was applied and incubated for 1 hour in the dark. Nuclei were stained with DAPI (1 μg/mL) for 10 minutes, and slides were mounted with anti-fade medium.

For cellular immunofluorescence, cells were fixed with 4% PFA, permeabilized with 0.25% Triton X-100, and blocked with 3% goat serum, followed by the same staining procedure as for tissue sections. Images were acquired using the LSM900 laser confocal microscope (Zeiss, Oberkochen, Germany) under appropriate laser channels.Quantitative analysis of immunofluorescence co-localization was performed using ImageJ software, and the percentage of dual-positive cells or the fluorescence intensity of target proteins was compared among groups.

### 2.11 SA-β-Gal assay

The SA-β-Gal staining was performed using Senescence β-Galactosidase Staining Kit (Beyotime, Shanghai, China). For tissue staining, liver biopsy tissue was embedded in OCT compound and sectioned at a thickness of 5 μm. The sections were fixed with the SA-β-Gal staining fixative for 10 minutes at room temperature. Cultured cells were fixed in 4% PFA for 15 minutes. After fixation, the sections or cells were washed three times with PBS (pH 7.4), each time for 5 minutes and subsequently incubated in SA-β-Gal staining solution at 37 °C overnight. Images were acquired using a light microscope (Axio Imager A2, Zeiss, Oberkochen, Germany).

### 2.12 Transcriptome sequencing analysis

Transcriptome sequencing was performed on the H_2_O_2_-induced senescent RAW264.7 macrophage model. Cells treated with H_2_O_2_ served as the experimental group, while PBS-treated cells were used as controls, with three biological replicates per group. Sequencing data analysis was carried out by NovelBio Bio-Pharm Technology Co., Ltd. (Shanghai, China). Total RNA was extracted using FastPure Cell/Tissue Total RNA Isolation Kit V2 (Vazyme, Nanjing, China), and RNA purity was assessed with a microspectrophotometer (OD260/280 ratio between 1.8-2.0); qualified samples were immediately transferred to liquid nitrogen for storage. Raw reads were processed with FastQC to remove adaptors and low-quality sequences, and the resulting clean reads were aligned to the reference genome (mm10_Ensembl) using STAR. Gene expression levels were quantified with HTSeq-count, and differential expression analysis was performed using DESeq2 or edgeR, with genes meeting |log_2_FC| > 1 and FDR < 0.05 considered differentially expressed. Functional enrichment analyses, including Gene Ontology (GO) and Kyoto Encyclopedia of Genes and Genomes (KEGG) pathway analysis, were conducted on the identified differentially expressed genes. In addition, Gene Set Enrichment Analysis (GSEA), which evaluates the enrichment of predefined gene sets across the entire ranked gene list without applying an arbitrary fold-change cutoff, was performed using the clusterProfiler package (version 4.0) in R. Genes were pre-ranked based on the log_2_(fold change) values, and pre-defined gene sets from the Molecular Signatures Database (MSigDB) were tested for enrichment. Normalized enrichment scores (NES) and FDR < 0.25 were used to determine statistically significant enrichment.

### 2.13 Lipid droplet staining

Cultured cells were washed with PBS and fixed with 4% PFA for 10 minutes. After washing with PBS, cells were stained with 2.5 μmol/L BODIPY493/503 (MCE, New Jersey, USA) for 30 minutes in the dark. The cells were then washed three times with PBS and stained with DAPI for 30 minutes. Finally, the cells were observed using the LSM900 laser confocal microscope.

### 2.14 Determination of protein stability by cycloheximide

After H_2_O_2_ pre-treatment, RAW264.7 cells were treated with cycloheximide (CHX; MCE, New Jersey, USA) at a final concentration of 532.5 nmol/L. Cells were collected at 0, 2, 4, 6 and 8 hours. Total protein was extracted, and FASN expression was assessed by Western blotting.

### 2.15 MG132 and chloroquine assays for protein stability

After H_2_O_2_ pre-treatment, RAW264.7 cells were treated with chloroquine (CQ; MCE, New Jersey, USA) at a final concentration of 40 μmol/L or MG132 (MCE, New Jersey, USA) at 1 μmol/L. The cells were collected after 2 hours. To further examine the degradation route of FASN, following the 2 hours pretreatment with CQ or MG132, CHX was added to the culture medium at a final concentration of 532.5 nmol/L, and the cells were incubated for an additional 6 hours. Subsequently, cells were harvested, total protein was extracted, and FASN protein levels were assessed by Western blotting.

### 2.16 FASN inhibitor treatment

After H_2_O_2_ pre-treatment, RAW264.7 cells were incubated with TVB-2640 (MCE, New Jersey, USA) at 0.052 μmol/L, GSK2194069 (MCE, New Jersey, USA) at 7.7 nmol/L, or C75 (MCE, New Jersey, USA) at 35 μmol/L for 48 hours, followed by cell collection and total protein extraction.

### 2.17 Statistical analysis

Statistical analysis was performed using GraphPad Prism 9. Comparisons between two groups were performed using the Mann-Whitney U test. For comparisons involving three or more groups, one-way analysis of variance (ANOVA) followed by Tukey’s *post hoc* test was applied. Wilcoxon signed-rank test or paired t-test was used for paired samples as appropriate. All data were expressed as mean ± standard deviation (SD). Spearman’s test was used for correlation analysis. A p-value below 0.05 was considered statistically significant.

## 3 Results

### 3.1 Cellular senescence is present in a mouse model of hepatic fibrosis

**Figure 1:**
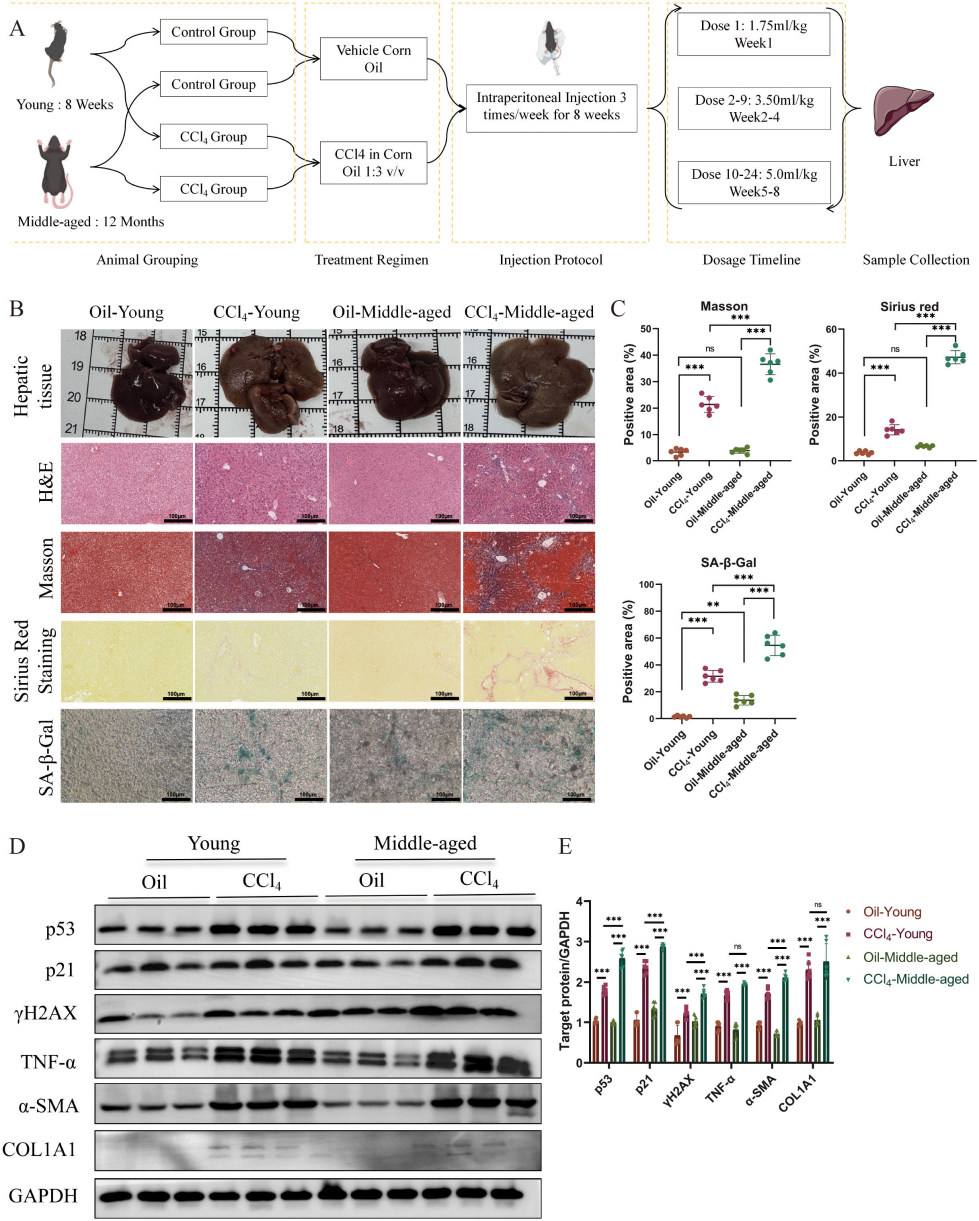
Detection of cellular senescence in fibrotic mouse liver tissues. Senescence-associated indicators were evaluated using histological staining and western blotting. **(A)** Schematic diagram illustrating the establishment of the hepatic fibrosis model in male C57BL/6 mice (n = 6 per group). **(B)** Representative histological images of liver sections from the indicated groups (Oil-Young, CCl_4_-Young, Oil-Middle-aged, CCl4-Middle-aged, n = 6 per group). H&E, Masson’s trichrome, Sirius Red, and senescence-associated β-galactosidase (SA-β-gal) staining were performed to assess histopathological changes and senescence phenotypes. **(C)** Quantitative analysis of SA-β-Gal-positive staining was performed, and collagen deposition was quantified on Masson’s trichrome- and Sirius Red-stained sections in liver tissues from each group (n = 6 per group). **(D)** Western blot analysis of senescence-associated proteins in liver tissues from the indicated groups (n = 6 per group). **(E)** Densitometric quantification of senescence-associated proteins in liver tissues from each group, as determined by western blotting (n = 6 per group). Statistical comparisons between two groups were performed using the Mann–Whitney U test. For comparisons involving three or more groups, one-way analysis of variance (ANOVA) followed by Tukey’s *post hoc* test was applied. **P < 0.01, ***P < 0.001; ns, not significant.

To investigate the presence of cellular senescence in hepatic fibrosis, we established a mouse model using C57BL/6 mice. Young (8-week-old) and middle-aged (12-month-old) mice were subjected to intraperitoneal injections of 25% CCl_4_ (v/v in corn oil), with doses progressively adjusted based on injection frequency and body weight (**Figure 1A**). During the modeling period, a significant decline in body weight was observed in all CCl_4_-treated groups, which was markedly more pronounced in middle-aged mice compared to young CCl_4_-treated mice (*P* < 0.001) (**Supplementary Figure 1A**). After modeling, serum biochemical analysis revealed levels of ALT, AST, and ALP were significantly higher in the middle-aged CCl_4_-treated mice compared to young CCl_4_-treated mice (*P* < 0.001). In contrast, no significant changes between these two groups were observed in TBIL levels, DBIL or γ-GT (*P* > 0.05) (**Supplementary Figure 1B**).

Gross examination and histopathological staining of liver tissues were performed. H&E staining confirmed greater inflammatory cell infiltration in middle-aged CCl_4_-treated mice compared to the young CCl_4_-treaded mice. Masson’s trichrome staining demonstrated more extensive blue collagen deposits and markedly thickened fibrous septa in middle-aged versus young CCl_4_-treated mice. Similarly, Sirius Red staining revealed enhanced deposition of fibrillar collagen in the livers of middle-aged CCl_4_-treated mice relative to young CCl_4_-treated mice (**Figure 1B**). Quantitative analysis of collagen area (%) confirmed a significant increase in the middle-aged CCl_4_ group relative to the young CCl_4_ group (*P* < 0.001) (**Figure 1C**).

Furthermore, senescence-associated SA-β-Gal staining of liver cryosections showed that abundant positive staining was present in middle-aged CCl_4_-treated mice and was predominantly localized to fibrotic areas, indicating a high burden of senescent cells in middle-aged fibrotic livers (*P* < 0.01) (**Figures 1B, C**).

Gene expression analysis in liver tissue showed significant upregulation in the CCl_4_ group of senescence-associated genes (*p19*, *p21*, *p53*, *Rage*, *Igfbp7*), SASP-related chemokines (*Ccl2*, *Ccl5*, *Cxcl10*, *Angptl2*), the key SASP regulator (*Serpine1* also known as *Pai*-1), matrix metalloproteinases (*Mmp3*, *Mmp13*), pro-inflammatory cytokines (*Il-1α*, *Il-1β*, *Il-6*, *Tnf-α*, *Tgf-β*), and hepatic fibrosis markers (*α-sma*, *Col1a1*). Notably, when comparing the two age cohorts, middle-aged mice subjected to CCl_4_ challenge exhibited further elevated transcript levels of *p19*, *p21*, *p53*, *α-sma*, *Col1a1*, *Ccl2*, *Ccl5*, *Cxcl10*, *Tgf-β*, *Il-1β*, *Il-6*, *Tnf-α*, *Igfbp7*, and *Mmp13* relative to their young CCl_4_-treated counterparts, indicating an age-dependent exacerbation of both cellular senescence and fibrotic responses (*P* < 0.01) (**Supplementary Figure 1C**).

Western blot analysis corroborated these findings, showing increased protein levels of senescence and DNA damage-related marker (p21, p53, γH2AX), the pro-inflammatory cytokines TNF-α, and fibrotic markers (α-SMA, COL1A1) (*P* < 0.001) (**Figures 1D, E**). Notably, compared with young CCl_4_-treated mice, middle-aged CCl_4_-treated mice exhibited further significant elevations in p21, p53, γH2AX, and α-SMA protein levels (*P* < 0.001), whereas TNF-α and COL1A1 showed an upward trend that did not reach statistical significance (*P* > 0.05). Collectively, these data confirmed the presence of cellular senescence in fibrotic livers, with more pronounced senescence and certain fibrotic features in middle-aged CCl_4_-treated mice.

### 3.2 Macrophages undergo cellular senescence in CCl_4_-induced hepatic fibrosis

**Figure 2:**
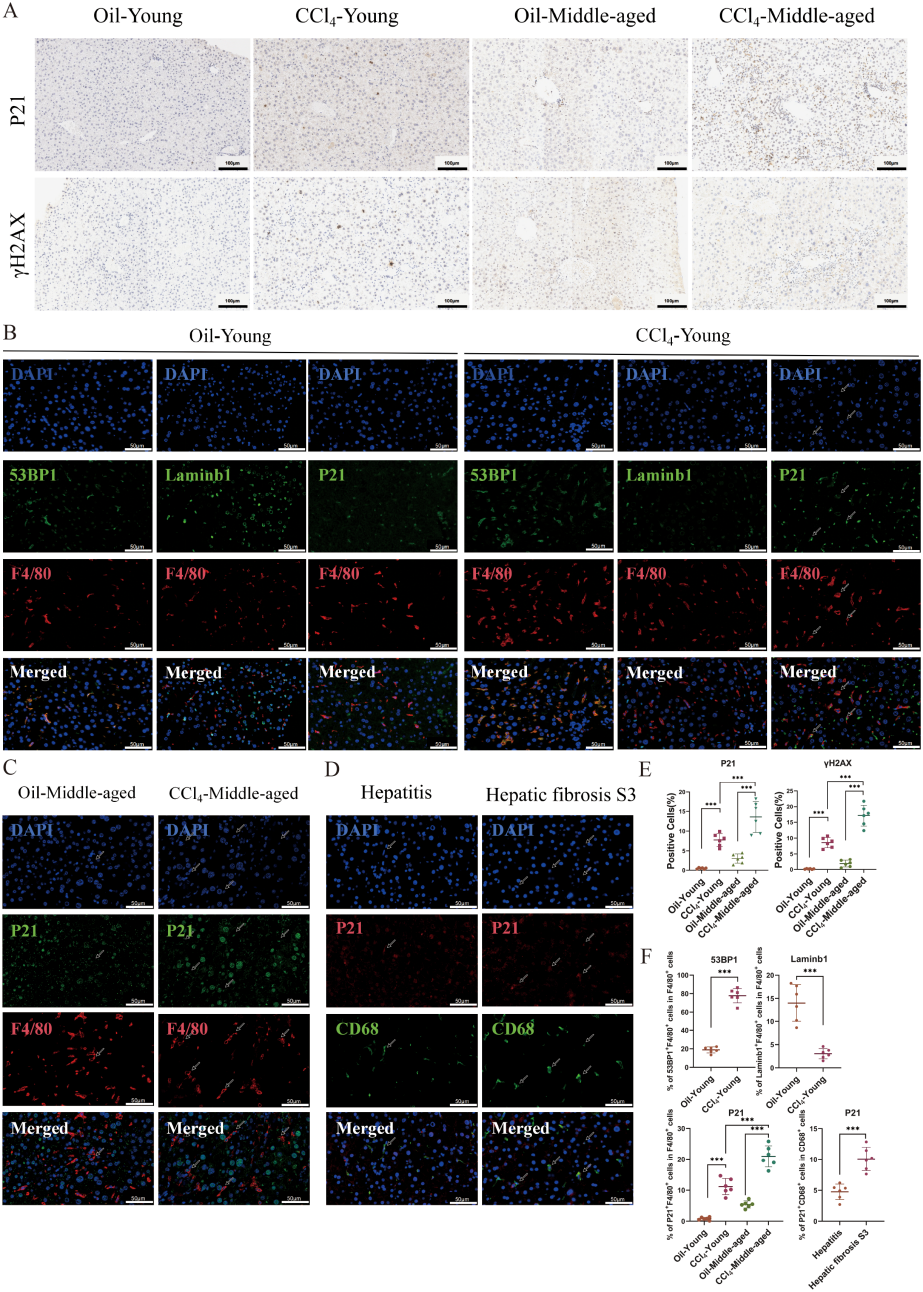
Increased cellular senescence in macrophages during liver fibrogenesis is further enhanced in middle aged mice. **(A)** Immunohistochemical staining for P21 and γH2AX in liver tissues from the indicated groups of mice. **(B)** Immunofluorescence double staining showing the colocalization of senescence-associated markers (53BP1, Laminb1, P21) with the macrophage marker F4/80 in liver tissues from Oil-Young and CCl4-Young mice. **(C)** Immunofluorescence double staining for P21 and F4/80 in liver tissues from Oil-Middle aged and CCl4-Middle aged mice. **(D)** Immunofluorescence co-staining for P21 and the macrophage marker CD68 in liver tissues from patients with hepatitis and liver fibrosis S3. **(E)** Quantification of immunohistochemical positive signals in mouse liver tissues from each group (n = 6 per group). **(F)** Quantification of immunofluorescence double-positive cells in mouse and human liver tissues from the indicated groups (n = 6 per group). Statistical comparisons between two groups were performed using the Mann–Whitney U test. For comparisons involving three or more groups, one-way analysis of variance (ANOVA) followed by Tukey’s *post hoc* test was applied. ***P < 0.001; ns, not significant.

Previous studies have documented the occurrence of cellular senescence in hepatocytes and HSCs during liver fibrogenesis (17–19). To determine whether additional cell types undergo senescence in the CCl_4_-induced fibrosis model, we performed immunohistochemical and immunofluorescence staining. Immunohistochemical analysis revealed positive staining for p21 and γH2AX in fibrotic liver tissue from CCl_4_-treated mice, with significantly enhanced immunoreactivity observed in middle-aged mice relative to their young counterparts (*P* < 0.001) (**Figures 2A, E**).

The spatial distribution of these senescence markers suggested that immune cells within the fibrotic liver may also undergo senescence. Considering that macrophages represent a predominant population of liver-resident immune cells and are critically involved in hepatic immunity and tissue repair, we therefore focused on investigating whether macrophage senescence occurred in the context of CCl_4_-induced liver fibrosis. Immunofluorescence analysis in young mice demonstrated increased 53BP1 foci formation and diminished Laminb1 immunoreactivity in F4/80^+^ macrophages following CCl_4_ treatment compared to oil-treated controls (*P* < 0.001) (**Figures 2B, F**). To further examine the age-dependent effect, dual immunofluorescence staining for p21 and F4/80 was performed in middle-aged mice. It should be noted that in the fibrotic regions of middle-aged CCl_4_-treated livers, the overall F4/80^+^ immunofluorescent area appeared reduced compared to that in oil-treated controls (**Figure 2C**). This reduction likely reflects a decline in total tissue cellularity and the physiological attrition of embryonically-derived Kupffer cells during aging, rather than a specific downregulation of macrophage abundance (2, 20). Importantly, despite the decreased total F4/80^+^ area, a significantly higher proportion of p21^+^F4/80^+^ co-localization was observed in middle-aged CCl_4_-treated mice compared with oil-treated controls (*P* < 0.001) (**Figures 2C, F**). Quantitative comparison between the two CCl_4_-treated cohorts confirmed that the proportion of p21^+^F4/80^+^ cells were significantly greater in middle-aged mice than in young mice (*P* < 0.001) (**Figure 2F**).

Finally, analysis of clinical samples from patients with hepatitis and liver fibrosis S3 similarly demonstrated a significantly increased proportion of p21^+^CD68^+^ cells in liver fibrosis S3 (*P* < 0.001) (**Figures 2D, F**). These data establish that macrophages acquire a senescent phenotype in fibrotic livers.

### 3.3 Establishment of a senescence model in RAW264.7 murine monocytic macrophages

**Figure 3:**
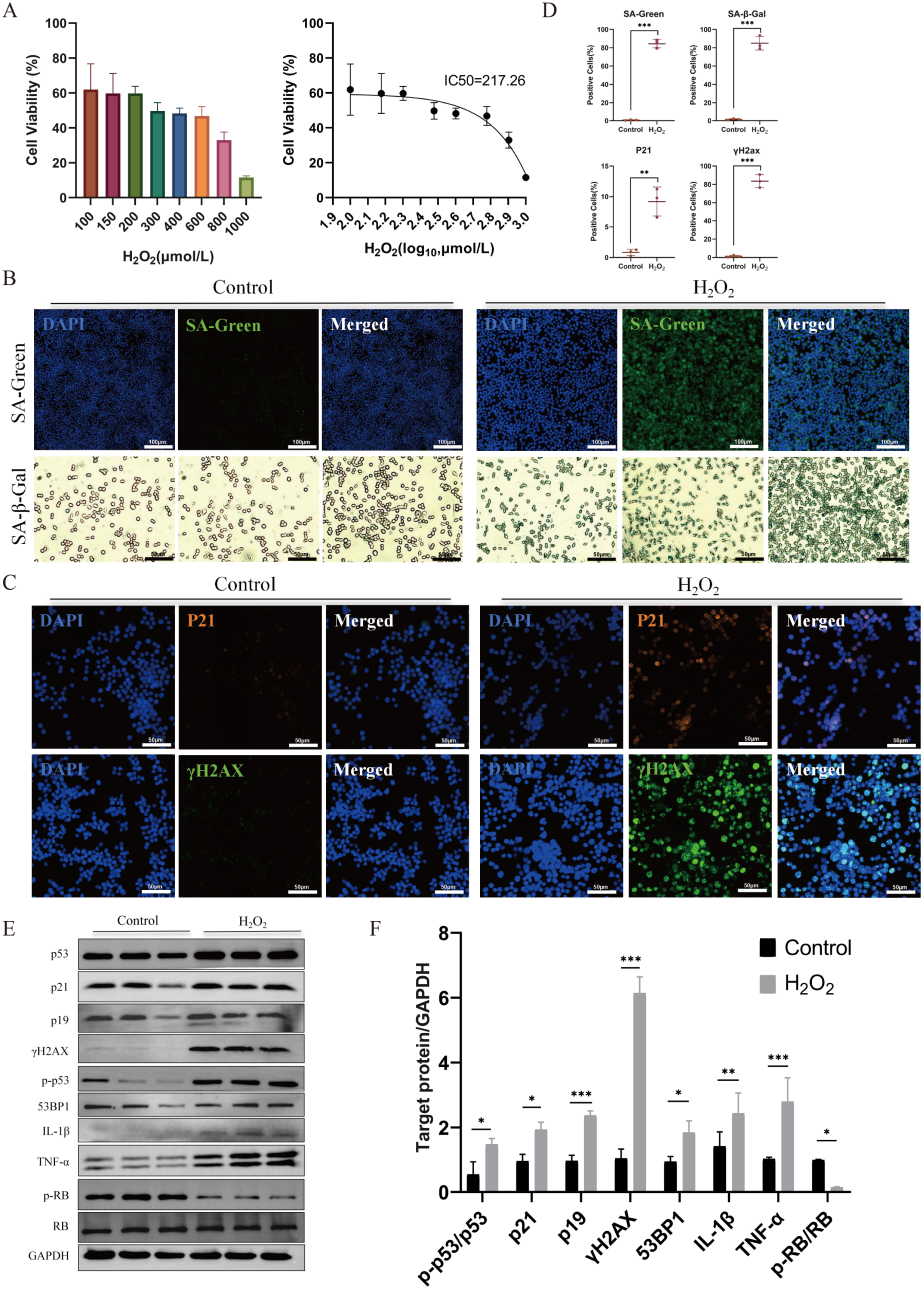
Establishment of a senescent macrophage model using RAW264.7 cells. **(A)** Cell viability was assessed by CCK-8 assay in RAW264.7 cells treated with various concentrations of H_2_O_2_ to determine the optimal concentration for inducing senescence. **(B)** Representative images showing SA-Green and SA-β-gal staining in the control and H_2_O_2_ groups (n = 3). **(C)** Representative immunofluorescence images depicting p21 and γH2AX expression in the control and H_2_O_2_ groups (n = 3). **(D)** Quantitative analysis of senescence-associated indicators in the control and H_2_O_2_-treated groups (n = 3). **(E)** Western blot analysis of protein expression related to senescence, DNA damage, and SASP components in the control and H_2_O_2_ groups (n = 3). **(F)** Quantitative analysis of the Western blot results, showing the relative expression levels of senescence, DNA damage, and SASP related proteins (n = 3). Comparisons between two groups were performed using the Mann-Whitney U test. *P < 0.05, **P < 0.01, ***P < 0.001; ns, not significant.

To investigate the role and mechanism of macrophage senescence in hepatic fibrosis, we established an *in vitro* model by treating RAW264.7 murine macrophages with H_2_O_2_. A CCK-8 assay determined the half-maximal inhibitory concentration (IC_50_) of H_2_O_2_ for these cells to be 217.26 µmol/L (**Figure 3A**). Based on preliminary experiments and existing literature, a concentration of 200 µmol/L H_2_O_2_ for 2 hours was selected for model induction (21).

Cell senescence was further confirmed by multiple assays. SA-β-Gal staining and senescence-associated fluorescence intensity were significantly increased, and immunofluorescence staining for p21 and γH2AX showed elevated expression in the H_2_O_2_ group (*P* < 0.01) (**Figures 3B–D**). RT-qPCR analysis revealed upregulation of senescence, cell cycle arrest, and DNA damage-related genes (*p21*, *Rage*), SASP factors (*Il-1α*, *Il-1β*, *Il-6*, *Tnf-α*, *Tgf-β*), and MMPs (*Mmp3*, *Mmp13*), alongside downregulation of *H2ax*, *Hdac1*, and *Hmgb1* in treated cells (*P* < 0.05) (**Supplementary Figure 2A**).

Western blot analysis further confirmed the upregulation of senescence markers (p19, p21), DNA damage-related proteins (γH2AX and 53BP1), and pro-inflammatory cytokines (IL-1β and TNF-α). Additionally, the p-p53/p53 ratio was significantly increased, whereas the p-RB/RB ratio was significantly decreased in H_2_O_2_-treated cells relative to controls (*P* < 0.05) (**Figures 3E, F**). These changes are mechanistically concordant with the observed p21 upregulation and reflect concurrent activation of the p53–p21 senescence pathway and hypophosphorylation-dependent RB activation, reinforcing a stable senescent cell cycle arrest.

A wound healing assay demonstrated that H_2_O_2_ treatment significantly impaired cell migration compared to the control group at both 12 and 24 hours (*P* < 0.05) (**Supplementary Figures 2B, C**). Collectively, these data validate the successful establishment of an H_2_O_2_-induced senescence model in RAW264.7 macrophages. It is worth noting that the senescent state observed in H_2_O_2_ treated RAW264.7 cells aligns closely with the phenotype identified in hepatic macrophages from middle-aged fibrotic mice. Given this parallel, this model offered a suitable platform for subsequent mechanistic investigation of macrophage senescence in the context of liver fibrosis.

### 3.4 Senescent RAW264.7 macrophages promote the activation of hepatic stellate cells LX-2

**Figure 4:**
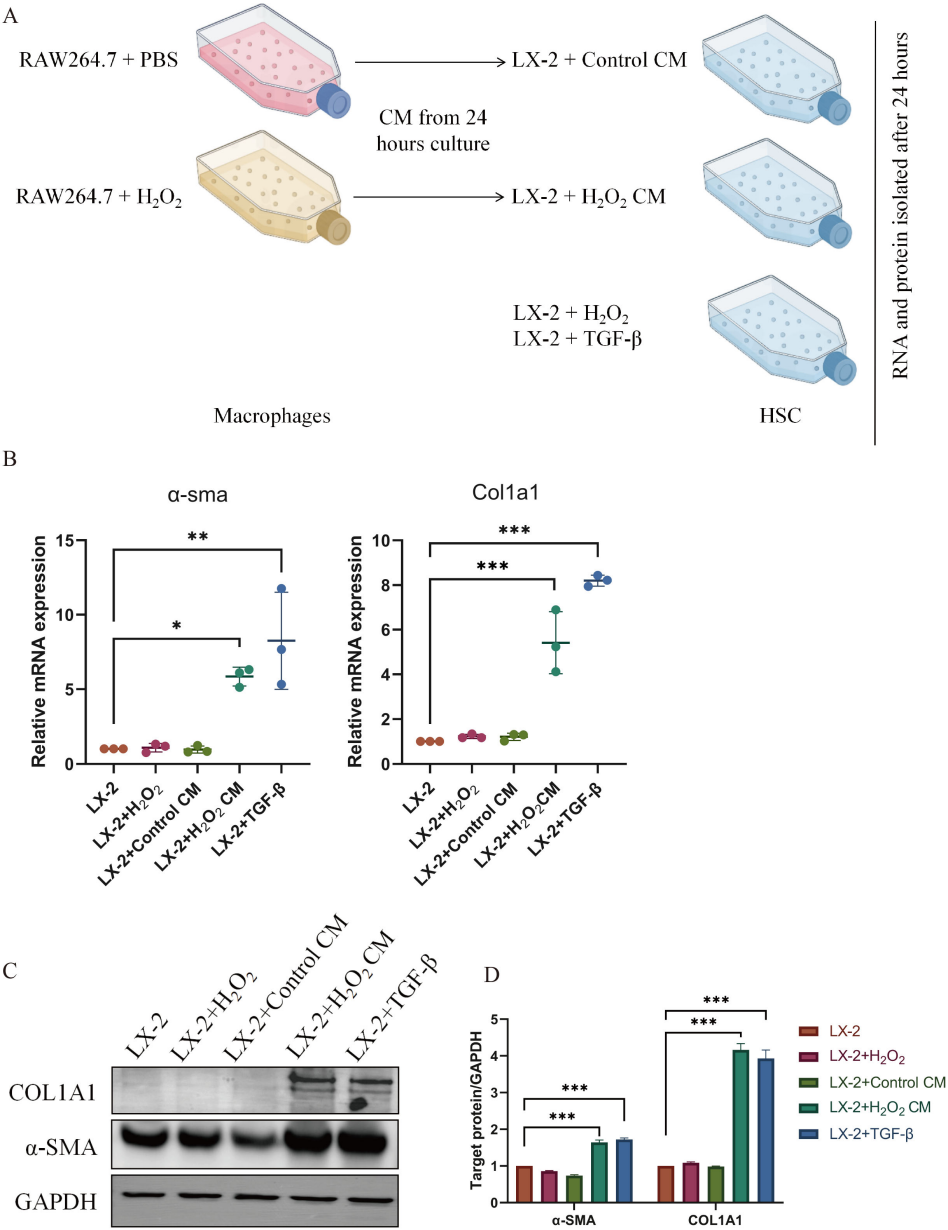
Effect of senescent macrophage conditioned medium on the activation of LX-2 cells. LX-2 cells were treated with H_2_O_2_, CM from control or H_2_O_2_-induced senescent RAW264.7 macrophages, with TGF-β treatment serving as a positive control. **(A)** flow chart of the co-culture of senescent RAW264.7 macrophages with LX-2 cells. **(B)** RT-qPCR analysis of *Col1a1* and *α-sma* mRNA expression levels in LX-2 cells from each group (n = 3). **(C)** Western blot analysis of COL1A1 and α-SMA protein expression in LX-2 cells from the indicated groups (n = 3). **(D)** Quantification of relative COL1A1 and α-SMA protein expression levels in LX-2 cells from each group (n = 3). Statistical comparisons between two groups were performed using the Mann–Whitney U test. For comparisons involving three or more groups, one-way analysis of variance (ANOVA) followed by Tukey’s *post hoc* test was applied. *P < 0.05, **P < 0.01, ***P < 0.001; ns, not significant.

Having established that macrophages undergo senescence in CCl_4_-induced fibrotic livers and that this phenotype is exacerbated in middle-aged mice, we next asked whether senescent macrophages functionally contribute to liver fibrosis progression. HSCs have been reported to be activated by various profibrotic and proinflammatory stimuli, including TGF-β, PDGF, and cytokines such as IL-1β and TNF-α, many of which are well-characterized SASP components (10). We therefore hypothesized that the SASP factors secreted by senescent macrophages may directly promote HSC activation. To test this hypothesis, we performed indirect co-culture experiments using conditioned medium (CM) collected from senescent RAW264.7 cells to treat LX-2 cells (**Figure 4A**). Both RT-qPCR and Western blot analyses demonstrated that CM from senescent macrophages significantly increased the mRNA and protein levels of α-SMA and COL1A1 in LX-2 cells compared to control CM (*P* < 0.05) (**Figures 4B–D**). Collectively, these results indicate that senescent macrophages possess a potent capacity to activate HSCs.

### 3.5 Transcriptomic profiling reveals senescence-associated signatures and fatty acid metabolic reprogramming in senescence macrophages

**Figure 5:**
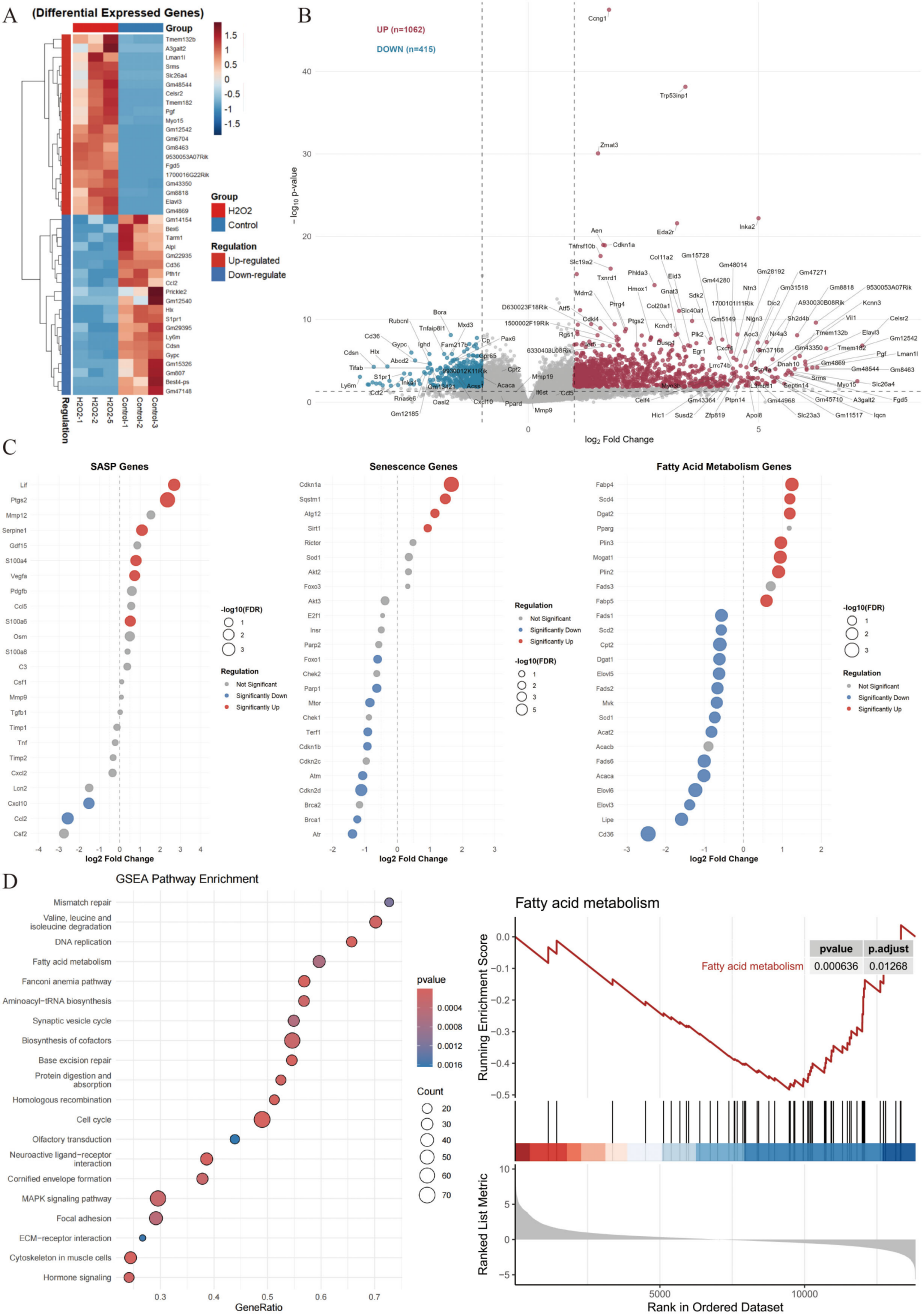
Transcriptomic profiling of RAW264.7 cells treated with or without H_2_O_2_. **(A)** Heatmap showing the expression patterns of differentially expressed genes (DEGs) between the control and H_2_O_2_-treated groups (|log_2_FC| > 1, FDR < 0.05). **(B)** Volcano plot illustrating the distribution of DEGs, with 1,062 upregulated and 415 downregulated genes identified (|log_2_FC| > 1, FDR < 0.05). **(C)** Bubble plot illustrating the overlap between differentially expressed genes (DEGs) and curated gene sets associated with the senescence-associated secretory phenotype (SASP), cellular senescence regulation, and fatty acid metabolism. **(D)** Gene Set Enrichment Analysis (GSEA) results showing enriched pathways (left panel) and representative running enrichment score plots with the corresponding rank in ordered dataset (right panel).

To investigate the potential mechanisms driving macrophage senescence, transcriptome sequencing was performed on the established H_2_O_2_-induced senescent RAW264.7 macrophage model, with PBS-treated control cells serving as the reference (n = 3). Transcriptomic analysis identified 1,477 significantly differentially expressed genes (DEGs) between senescent and normal macrophages, of which 1,062 genes were upregulated and 415 genes were downregulated (**Figures 5A, B**).

Analysis of the DEGs revealed that in senescent macrophages, the expression levels of activation markers (*Ntn3*, *Cxcr4*, *Hmox1*, *Kcnd1*, *Eda2r*), senescence markers (*Cdkn1a*, *Sqstm1*, *Atg12*), and genes specifically involved in fatty acid synthesis and lipid storage (*Fabp4*, *Scd4*, *Dgat2*) were significantly increased. Conversely, the expression levels of certain SASP chemokines (*Ccl2*, *Cxcl10*) and key regulators of fatty acid β-oxidation (*Cd36*, *Acaca*, *Cpt2*) were significantly decreased (**Figure 5C**). This coordinated shift, namely the suppression of fatty acid oxidation coupled with enhanced fatty acid synthesis, is consistent with the concept of metabolic reprogramming, a phenomenon increasingly recognized as a hallmark of senescent cells (22, 23). Gene Ontology (GO) and Kyoto Encyclopedia of Genes and Genomes (KEGG) enrichment analyses showed that the DEGs in senescent macrophages were significantly enriched in biological processes and signaling pathways related to cell cycle regulation, DNA damage response, ferroptosis, glycolysis, the p53 signaling pathway, and the NF-κB signaling pathway (**Supplementary Figures 3A–D**). Gene Set Enrichment Analysis (GSEA), which evaluates the enrichment of predefined gene sets across the entire ranked gene list without applying an arbitrary fold-change cutoff, further revealed that senescent macrophages exhibited significant enrichment in biological processes and signaling pathways related to cell cycle regulation, DNA replication, the mitogen-activated protein kinase (MAPK) signaling cascade, the focal adhesion kinase (FAK) signaling pathway, and fatty acid metabolism (**Figure 5D**). These findings raise the possibility that such reprogramming of fatty acid metabolism may contribute to the maintenance of the senescent phenotype and the functional alterations observed in senescent macrophages.

### 3.6 Senescent macrophages undergo fatty acid metabolic reprogramming to sustain the senescence phenotype and SASP secretion

**Figure 6:**
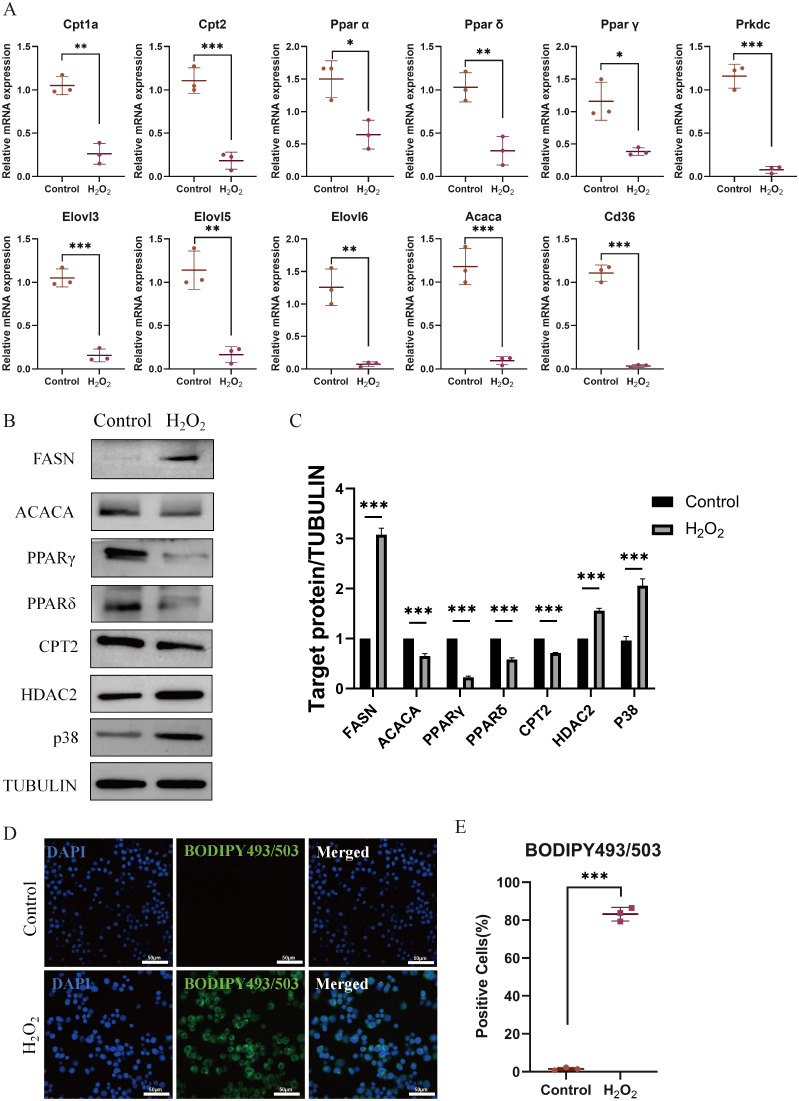
Senescent RAW264.7 macrophages exhibit fatty acid metabolic reprogramming and lipid accumulation. **(A)** RT-qPCR analysis of mRNA levels of genes involved in fatty acid β-oxidation in control and H_2_O_2_-induced senescent macrophages (n = 3). **(B)** Western blot analysis of protein expression related to fatty acid synthesis and fatty acid β-oxidation in the indicated groups (n = 3). **(C)** Quantification of the relative protein expression levels shown in **(B)** (n = 3). **(D)** BODIPY 493/503 lipid droplet staining in control and senescent macrophages. **(E)** Quantitative analysis of lipid droplet-positive areas (n = 3). Statistical comparisons between two groups were performed using the Mann-Whitney U test. *P < 0.05, **P < 0.01, ***P < 0.001; ns, not significant.

Based on the transcriptomic findings described above, we next sought to validate the interplay among fatty acid metabolism, inflammation, and cellular senescence. In agreement with the RNA-seq data, RT-qPCR analysis showed that senescent macrophages exhibited reduced expression of genes central to fatty acid β-oxidation, including the rate-limiting enzymes *Cpt1a* and *Cpt2*, as well as their upstream transcriptional regulators *Acaca*, *Ppar α*, *Ppar δ*, and *Ppar γ*. In addition, the fatty acid translocase *Cd36*, and the DNA damage response kinase *Prkdc* were also significantly diminished in senescent cells relative to controls (*P* < 0.05) (**Figure 6A**). Notably, the protein level of fatty acid synthase (FASN) was elevated, along with increases in histone deacetylase 2 (HDAC2) and p38 mitogen−activated protein kinase (p38), whereas the β-oxidation rate-limiting enzyme CPT2 and the transcriptional regulators ACACA, PPAR δ, and PPAR γ were consistently downregulated (*P* < 0.001) (**Figures 6B, C**).

BODIPY 493/503 staining for neutral lipids revealed significantly greater lipid droplet accumulation in H_2_O_2_-treated macrophages compared to controls (*P* < 0.001) (**Figures 6D, E**). Collectively, these results demonstrate that senescent macrophages undergo a metabolic shift characterized by promoted fatty acid synthesis and suppressed fatty acid β-oxidation, leading to intracellular lipid accumulation. This reprogrammed metabolic state may sustain the robust secretion of SASP factors and pro-inflammatory cytokines, thereby reinforcing the pro-fibrotic activity of senescent macrophages.

### 3.7 FASN protein degradation occurs via ubiquitination and autophagy pathways, and inhibition of FASN attenuates cellular senescence phenotypes

**Figure 7:**
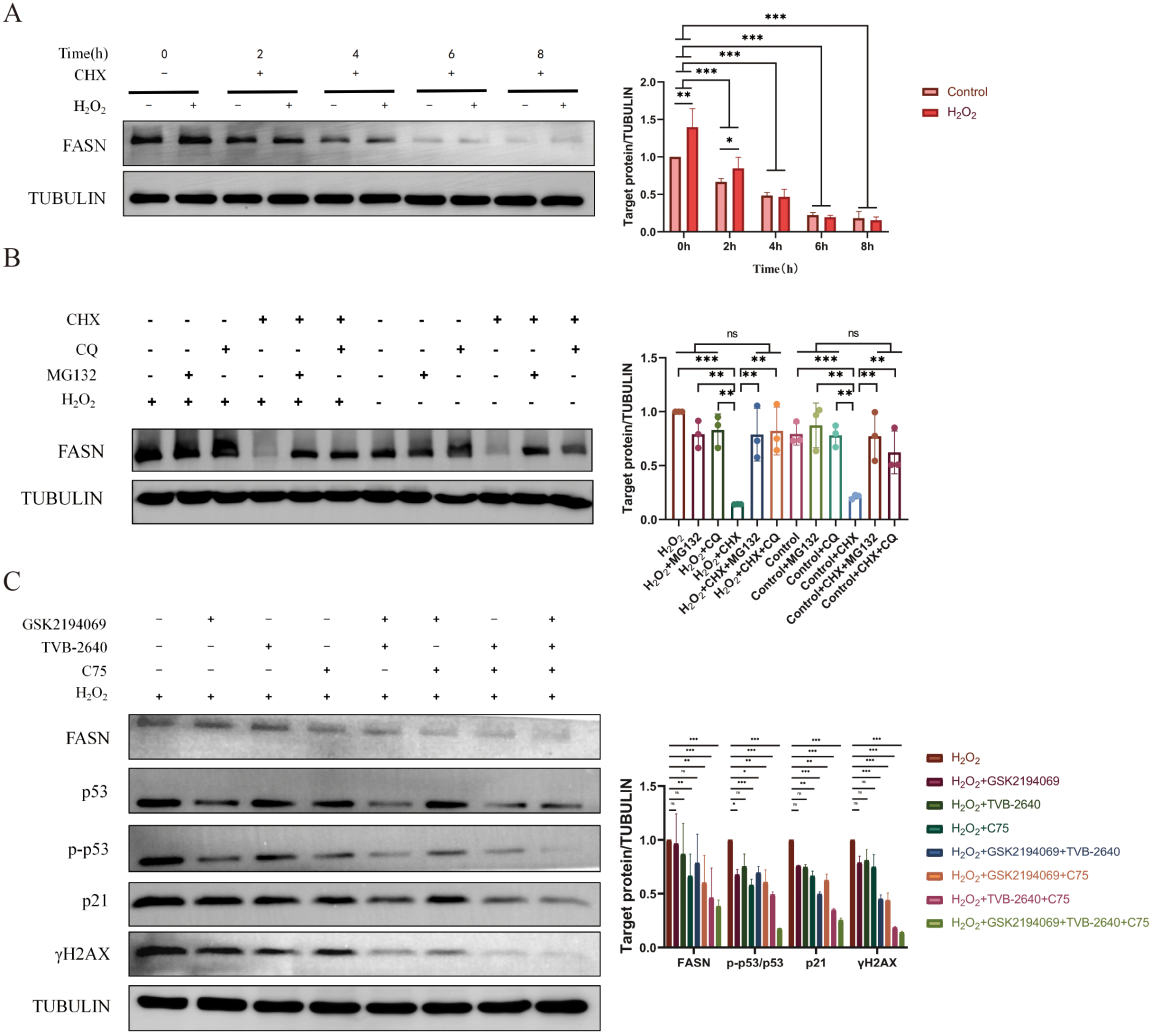
FASN protein is degraded via both ubiquitin-proteasome and autophagic pathways, and FASN inhibition attenuates the cellular senescence phenotype. **(A)** Western blot analysis of FASN protein levels at the indicated time points (0, 2, 4, 6, and 8 hours) following cycloheximide (CHX) treatment in control and H_2_O_2_-exposed cells, assessing FASN protein stability. **(B)** Western blot analysis of FASN degradation pathways in control and H_2_O_2_-treated cells. Cells were treated with CHX alone or in combination with the proteasome inhibitor MG132 or the autophagy inhibitor chloroquine (CQ), as indicated. **(C)** Western blot analysis of FASN and senescence-associated protein levels in H_2_O_2_-treated cells following treatment with different FASN inhibitors (GSK2194069, TVB-2640, C75) (n = 3), evaluating the effect of FASN inhibition on the senescent phenotype. Statistical comparisons between two groups were performed using the Mann–Whitney U test. For comparisons involving three or more groups, one-way analysis of variance (ANOVA) followed by Tukey’s *post hoc* test was applied. *P < 0.05, **P < 0.01, ***P < 0.001; ns, not significant.

Based on our above experimental findings, we next sought to investigate the regulatory mechanism underlying the altered FASN protein expression. Protein expression levels are governed by both synthesis and degradation pathways. To assess the stability of the FASN protein, we treated cells with CHX, an inhibitor of protein synthesis. Western blot results showed that FASN protein levels gradually decreased over time following CHX treatment (*P* < 0.01) (**Figure 7A**). Cellular protein degradation is predominantly mediated by two systems: the ubiquitin-proteasome pathway and the autophagic-lysosomal pathway. When senescent RAW264.7 macrophages were treated with the proteasome inhibitor MG132 or the autophagy inhibitor CQ, the degradation of FASN following CHX addition was attenuated, suggesting that both ubiquitination and autophagy pathways were involved in FASN protein turnover (*P* < 0.05) (**Figure 7B**). Quantitative analysis of band intensities was performed for all treatment groups, and statistical comparisons confirmed that the differences between the CQ/MG132 + CHX groups and the CQ/MG132 − CHX groups were not statistically significant (*P* > 0.05) (**Figure 7B**), indicating that the inhibitor pretreatment itself did not significantly alter basal FASN protein levels.

To further validate the functional importance of FASN in cellular senescence, we treated senescent RAW264.7 cells with three distinct FASN inhibitors (TVB-2640, GSK2194069, and C75). Western blot analysis revealed that all three inhibitors significantly reduced the expression of senescence markers (p21, γH2AX) and decreased the p-p53/p53 ratio compared with the H_2_O_2_-treated group (*P* < 0.05) (**Figure 7C**), indicating that FASN inhibition effectively attenuates the senescence-associated DNA damage response and p53 activation. Notably, among the three inhibitors, only C75 significantly reduced FASN protein levels, whereas TVB-2640 and GSK2194069 did not appreciably alter FASN protein abundance. The differential effect of C75 on FASN protein expression may be attributable to its additional pharmacological activity beyond enzymatic inhibition: C75 has been reported to suppress the expression of lipogenic genes, including Fasn, through inhibition of the SREBP-1 signaling pathway, and structural similarity between C75 and malonyl-CoA may further disrupt FASN protein stability (24, 25). Therefore, the reduction in senescence markers observed with all three inhibitors reflects their shared inhibition of FASN enzymatic activity, while the decrease in FASN protein itself is a C75-specific effect.

## 4 Discussion

This study aimed to elucidate the role of senescent macrophages in the development of liver fibrosis and their underlying metabolic regulatory mechanisms. Our results demonstrate that in a CCl_4_-induced liver fibrosis model, middle-aged mice exhibited more pronounced senescence and fibrotic features than young mice. Specifically, we found that macrophages acquire a senescent phenotype during liver fibrosis. Furthermore, these senescent macrophages displayed substantial reprogramming of fatty acid metabolism characterized by enhanced fatty acid synthesis, suppressed β−oxidation, and consequent intracellular lipid accumulation. Importantly, we further identified that the upregulation of FASN in these cells promoted the senescence phenotype, identifying FASN as a potential mediator that contributes to senescent macrophage dysfunction.

Cellular senescence is a stable form of cell cycle arrest triggered by diverse stressors, including telomere dysfunction, oncogenic activation, oxidative stress, and DNA damage, and is characterized by distinct morphological and metabolic alterations along with the acquisition of a complex secretome termed the SASP (26). Despite substantial advances in senescence research, no single universal marker unequivocally defines this heterogeneous cellular state; current identification therefore relies on combinatorial detection of multiple senescence-associated features, including cell cycle inhibitors, lysosomal SA-β-Gal activity, nuclear lamina alterations, persistent DNA damage signals and SASP secretion (27, 28). Pathologically, the aberrant accumulation of senescent cells creates a pro-inflammatory microenvironment that drives chronic tissue damage and contributes to disease pathogenesis. Therefore, to investigate this in the context of liver fibrosis, we established a CCl_4_-induced model in both young and middle-aged mice and assessed cellular senescence using a combination of the markers described above. Compared to controls, senescent phenotypes were more pronounced in the livers of young fibrotic mice and were further exacerbated in middle-aged fibrotic mice. Supporting our finding, aged livers have been reported to exhibit a higher incidence of cirrhosis and hepatocellular carcinoma (29).

Previously, studies of cellular senescence in liver fibrosis have primarily identified its presence in hepatocytes and HSCs, where it exerts context-dependent and often dichotomous effects on fibrogenesis (30, 31). Senescent hepatocytes accumulate with aging and metabolic stress, secrete a pro-inflammatory SASP, and promote HSC activation and fibrosis progression. Paradoxically, senescent HSCs can limit fibrogenesis by terminating myofibroblast proliferation and enhancing matrix degradation. This functional paradox illustrates the dualistic role of cellular senescence in liver homeostasis and disease (31–33). However, cellular senescence has also been documented within immune cells, including in the liver (13). In the present study, *in vivo* experiments revealed that macrophages in a CCl_4_-induced mouse model of liver fibrosis exhibit a significant senescent phenotype, characterized by upregulated expression of γH2AX, p21, and p53, along with decreased Laminb1 expression. It should be noted that the increased proportion of p21^+^F4/80^+^ macrophages were significantly greater in middle-aged CCl_4_-treated mice than in young CCl_4_-treated mice, providing direct quantitative evidence for age-dependent exacerbation of macrophage senescence. We did not systematically assess senescence in other non-parenchymal cell populations; however, previous work has documented that hepatic stellate cells, endothelial cells, and Kupffer cells can all enter a senescent state during chronic liver injury (21, 30). Thus, macrophage senescence represents one component of the broader senescence landscape in the fibrotic liver. In line with this observation, a recent study employing a p16INK4a−based lineage tracing system demonstrated that macrophages represent a compartment of senescent cells during liver fibrosis, and their selective clearance attenuated hepatocellular damage and fibrotic progression (15).

HSCs and macrophages activate each other through cytokines, such as TGF-β, IL-1β, CCL2, and CCL5, thereby driving ECM deposition, HSC activation, and fibrosis progression (34). However, the impact of senescent macrophages on HSC function has not been reported. Our *in vitro* experiments confirmed that senescent macrophages promoted HSC activation via paracrine pathways, as conditioned medium from these cells upregulated the expression of α-SMA and COL1A1 in LX-2 cells. Moreover, we found that senescent macrophages exhibit reduced migratory capacity, which might impair their timely recruitment to sites of injury for debris clearance, potentially exacerbating liver fibrosis. Nevertheless, this hypothesis requires further validation.

Furthermore, we found that senescence reprogrammed macrophage lipid metabolism toward decreased β-oxidation and increased fatty acid synthesis, leading to lipid accumulation. Specifically, we observed the expression of CPT1A and CPT2, two key enzymes in β-oxidation, was significantly reduced. This finding aligns with previous reports (35, 36). Moreover, the reduction of β-oxidation enzymes impairs mitochondrial function, which in turn accelerates cellular senescence (23). The expression of PPAR α, δ, and γ, which are key transcription factors for β-oxidation enzymes, was also markedly decreased in senescent macrophages. This observation is consistent with our expectations, as these nuclear receptors are known regulators of lipid metabolism and inflammation. However, we found that the expression of the lipid uptake receptor CD36 was significantly decreased. This result is inconsistent with a previous study showing that CD36 expression promotes cellular senescence (37). We tend to attribute this discrepancy to cellular heterogeneity.

The role of FASN, the key enzyme responsible for *de novo* palmitate synthesis, in cellular senescence exhibits marked cell-type and context specificity. For instance, FASN upregulation has been reported to promote senescence in HSCs and human dermal fibroblasts, a process attributed to lipid accumulation-induced oxidative stress (25). Conversely, in aged mouse adipose tissue, SPATA4-mediated FASN induction facilitates adipocyte differentiation, reduces inflammation, and alleviates age-related metabolic dysfunction (38). In our study, we observed an upregulation of FASN protein in senescent macrophages, and further demonstrated that this elevated expression actively contributes to macrophage senescence. Moreover, we found that FASN underwent dual degradation via both the ubiquitin-proteasome and autophagy pathways in these cells. This finding has not been previously reported in senescent macrophages, and further in-depth investigation is required to delineate the molecular machinery governing this dual degradation mechanism.

This study has several limitations. First, the number of clinical liver specimens was limited, which restricts the clinical validation of our findings. Second, mice older than 18 months were not used; future studies will employ aged mice (≥18 months) to better capture the effect of chronological aging on macrophage senescence. Third, FASN was not genetically manipulated (e.g., by knockdown or overexpression) to establish its causal role in the senescence phenotype and to validate the upstream and downstream regulatory mechanisms; future investigations will adopt these genetic approaches to further dissect the underlying pathways. Despite these limitations, our findings provide novel insights into the metabolic dimension of macrophage senescence in liver fibrosis and lay a foundation for future mechanistic and translational studies.

In summary, this study yields three principal findings. First, we demonstrate that macrophage senescence is a prominent feature of middle-aged fibrotic livers, with exacerbated expression of senescence markers and SASP components in middle-aged mice subjected to CCl_4_ challenge. Second, we show that senescent macrophages actively promote the activation of HSCs, thereby contributing to the progression of liver fibrosis. Third, we identify fatty acid metabolic reprogramming in senescent macrophages, specifically the upregulation of FASN coupled with suppressed fatty acid β-oxidation, as a key mechanism that sustains the senescence phenotype and pro-fibrotic activity. Collectively, these results identify FASN as a critical driver of macrophage senescence, providing a potential therapeutic target for liver fibrosis.

## Data Availability

The datasets presented in this study can be found in online repositories. The names of the repository/repositories and accession number(s) can be found below: PRJNA1458553 (Bioproject, NCBI).
