# Supplementary material for: Upregulated FASN-mediated lipogenesis in senescent macrophages contributes to liver fibrosis progression
Source: Front Immunol. 2026 Jul 14;17:1863063. doi: 10.3389/fimmu.2026.1863063 (PMC13407210; doi:10.3389/fimmu.2026.1863063)
Supplement: Supplementary file 1 [file Supplementaryfile1.docx]

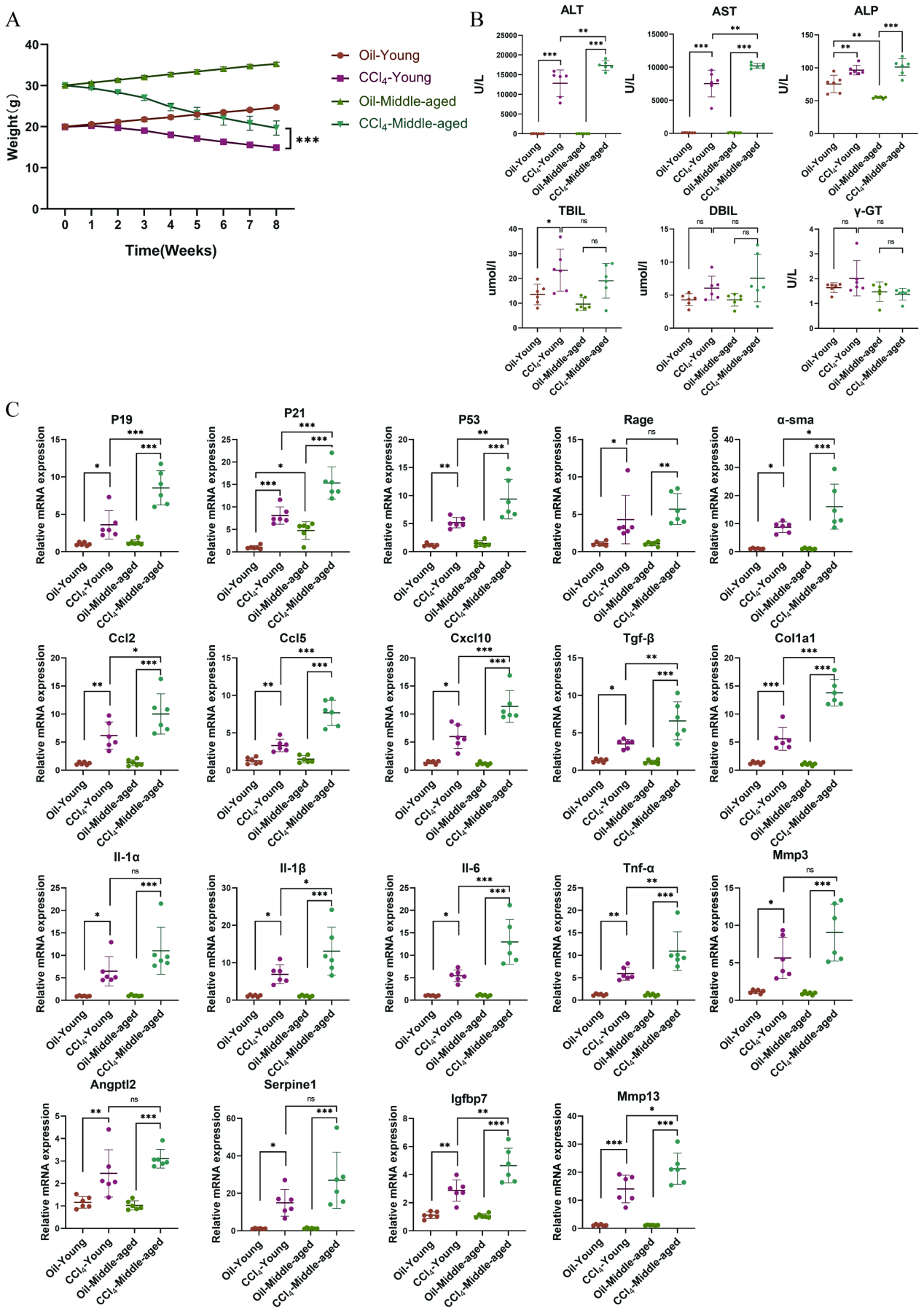


**Supplementary Figure 1. Serum biochemical parameters and hepatic mRNA expression levels in mice.** (A) Dynamic changes in body weight of mice during CCl_4_ treatment (n = 6 per group). (B) Serum levels of liver function-related biochemical parameters in the indicated groups (n = 6 per group). (C) The mRNA expression levels of target genes in liver tissues were determined by RT-qPCR (n = 6 per group). Statistical comparisons between two groups were performed using the Mann–Whitney U test. For comparisons involving three or more groups, one-way analysis of variance (ANOVA) followed by Tukey's post hoc test was applied. **P* < 0.05, ***P* < 0.01, ****P* < 0.001; ns, not significant.


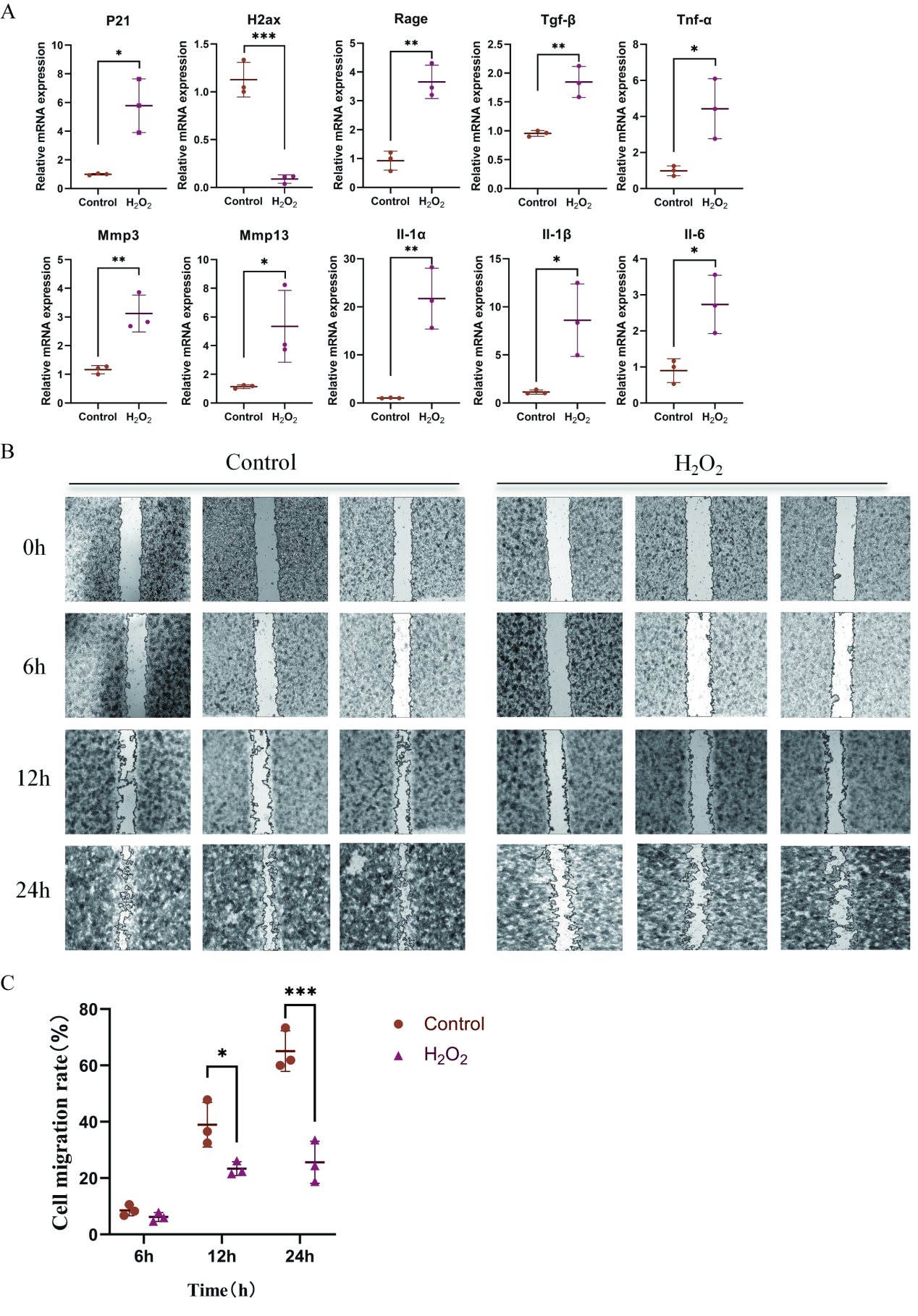


**Supplementary Figure 2. Changes in mRNA expression levels of senescence-associated genes and migratory capacity in a RAW264.7 cell senescence model.** (A) RT-qPCR analysis of mRNA levels of senescence-associated genes, DNA damage response genes, SASP components, and MMPs in RAW264.7 cells following H₂O₂ treatment (n = 3). (B) Wound healing assays assessing the migratory capacity of RAW264.7 cells after H₂O₂ treatment (n = 3). (C) Quantitative analysis of wound healing assays showing statistical comparisons of cell migration rates at different time points (6, 12, and 24 hours) across groups. Statistical comparisons between two groups were performed using the Mann-Whitney U test. **P* < 0.05, ***P* < 0.01, ****P* < 0.001; ns, not significant.


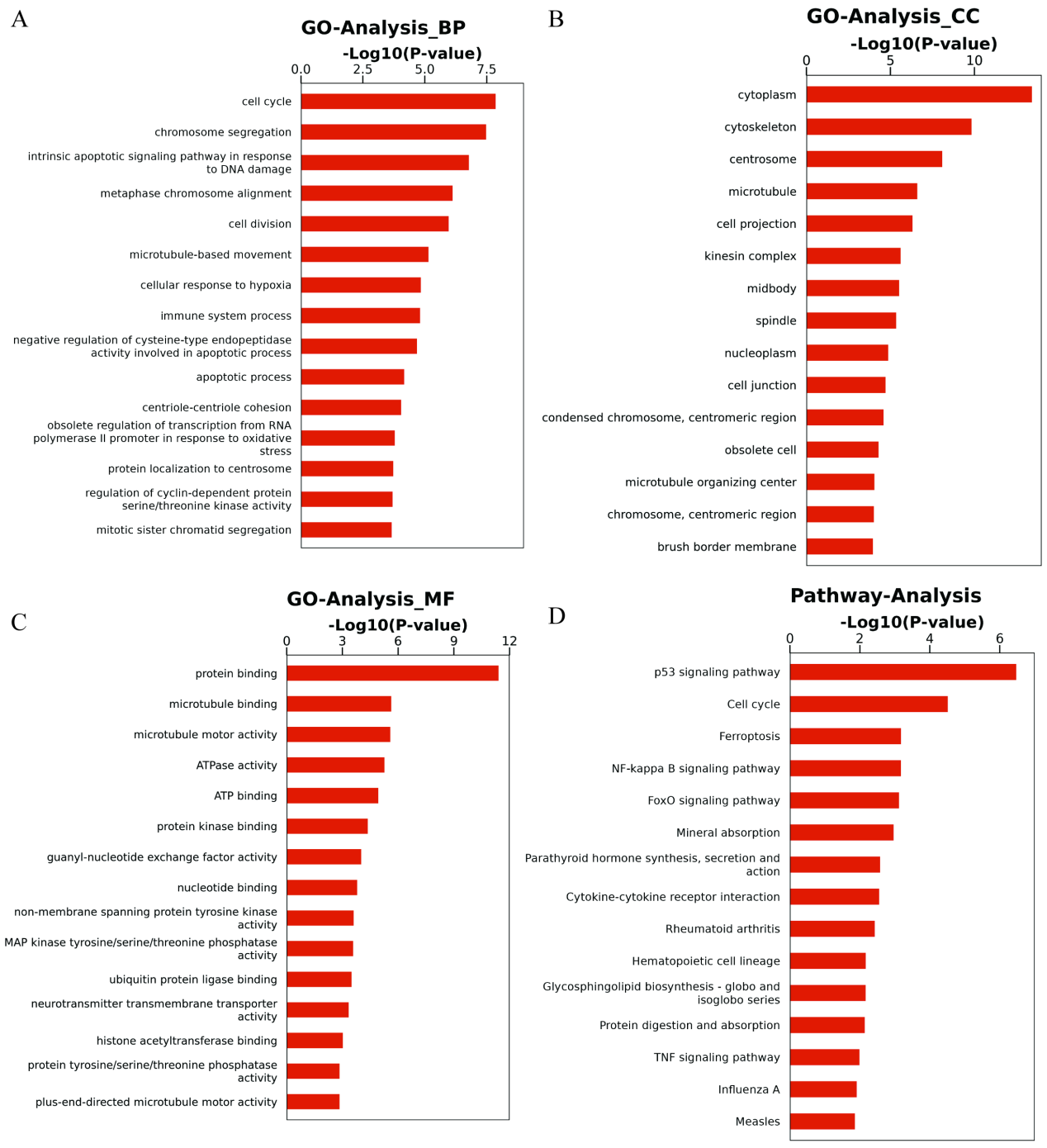


**Supplementary Figure 3. Transcriptomic profiling of RAW264.7 cells treated with or without H₂O₂.** (A) Gene Ontology Biological Process (GO-BP) enrichment analysis of differentially expressed genes (DEGs). (B) Gene Ontology Cellular Component (GO-CC) enrichment analysis of DEGs. (C) Gene Ontology Molecular Function (GO-MF) enrichment analysis of DEGs. (D) Kyoto Encyclopedia of Genes and Genomes (KEGG) pathway enrichment analysis of DEGs.

**Supplementary Table 1.** Primers for RT-qPCR of Mouse

| Gene | Forward primer（5’→3’） | Reverse primer（5’→3’） |
| --- | --- | --- |
| *Gapdh* | AGGTCGGTGTGAACGGATTTG | GGGGTCGTTGATGGCAACA |
| *P19* | CGCAAGGAAAGGAGGGAGGT | CTTGCCAAAGCGGTTCAGG |
| *P21* | TGAATGGAGACAGAGACCCC | GGGACCGAAGAGACAACGG |
| *P53* | GGGCTGAGACACAATCCTCC | TCATTGTAGGTGCCAGGGTC |
| *Rage* | CGGATTGGAGAGCCACTTGT | GAGGACCTTCCAAGCTTCAGT |
| *α-sma* | TCAGGGAGTAATGGTTGGAATG | CCCTCTCTTGCTCTGGGCTTCATC |
| *Col1a1* | GAAACCCGAGGTATGCTTGA | CATTGCACGTCATCGCACACAGC |
| *Ccl2* | CCAACTCTCACTGAAGCCAGCTCT | TCAGCACAGACCTCTCTCTTGAGC |
| *Ccl5* | ATATGGCTCGGACACCACTC | ACTTGGCGGTTCCTTCGAG |
| *Cxcl10* | GGTCTGAGTCCTCGCTCAAG | GTCGCACCTCCACATAGCTT |
| *Il-1α* | ACGTCAAGCAACGGGAAGAT | AAGGTGCTGATCTGGGTTGG |
| *Il-1β* | GAAATGCCACCTTTTGACAGTGAT | GGAAGGTCCACGGGAAAGAC |
| *Il-6* | TAGTCCTTCCTACCCCAATTTCC | TTGGTCCTTAGCCACTCCTTC |
| *Tnf-α* | CCTCTTCTCATTCCTGCTTGT | TGGGAACTTCTCATCCCTTTG |
| *Tgf-β* | CCTCGAGACAGGCCATTTGT | GACCAAGGCCAGCTGACTG |
| *Mmp3* | GTTCTGGGCTATACGAGGGC | TTCTTCACGGTTGCAGGGAG |
| *Mmp13* | CAGTTGACAGGCTCCGAGAA | TTCACCCACATCAGGCACTC |
| *Angptl2* | GCGACTCCTTTACCTGGCACAA | GTTGGAGTGAGCACAGGCGTTA |
| *Serpine1* | CTCCAAGGGGCAACGGATAG | AAGCAAGCTGTGTCAAGGGA |
| *Igfbp7* | GAAAGAAGGCAAGGAGGCGT | CATGTTGGCTTGTGGGCAAC |
| *H2ax* | GGTCAGAGAGACGCTTACCG | TTGTAGTTGAGTCGCTGGGG |
| *Cpt1a* | CTCCGCCTGAGCCATGAAG | CACCAGTGATGATGCCATTCT |
| *Cpt2* | CAGCACAGCATCGTACCCA | TCCCAATGCCGTTCTCAAAAT |
| *Ppar α* | AGAGCCCCATCTGTCCTCTC | ACTGGTAGTCTGCAAAACCAAA |
| *Ppar δ* | TCCATCGTCAACAAAGACGGG | ACTTGGGCTCAATGATGTCAC |
| *Ppar γ* | CTCCAAGAATACCAAAGTGCGA | GCCTGATGCTTTATCCCCACA |
| *Elovl3* | TTCTCACGCGGGTTAAAAATGG | GAGCAACAGATAGACGACCAC |
| *Elovl5* | ATGGAACATTTCGATGCGTCA | GTCCCAGCCATACAATGAGTAAG |
| *Elovl6* | GAAAAGCAGTTCAACGAGAACG | AGATGCCGACCACCAAAGATA |
| *Prkdc* | AAACCTGTTCCGAGCTTTTCTG | TAAGGGCACAGCATATCGCTT |
| *Cd36* | ATGGGCTGTGATCGGAACTG | TTTGCCACGTCATCTGGGTTT |
| *Acaca* | ATGGGCGGAATGGTCTCTTTC | TGGGGACCTTGTCTTCATCAT |

**Supplementary Table 2.** Primers for RT-qPCR of Human

| Gene | Forward primer（5’→3’） | Reverse primer（5’→3’） |
| --- | --- | --- |
| GAPDH | CGGGAAACTGTGGCGTGAT | GTCGCTGTTGAAGTCAGAGGAGA |
| α-SMA | GGTGATGGTGGGAATGG | CAGGGTGGGATGCTCTT |
| COL1A1 | AAGGTGTTGTGCGATGACG | GTTTCTTGGTCGGTGGGTG |

**Supplementary Table 3.** Patient Clinical Characteristics Table

| Pathology number | Gender | Age (years) | Clinical diagnosis | Pathological GS staging |
| --- | --- | --- | --- | --- |
| F20250029 | man | 49 | Chronic hepatitis B virus | G1 S1 |
| F20231363 | man | 53 | Chronic hepatitis B virus | G1 S1 |
| F20240874 | man | 53 | Chronic hepatitis B virus | G1 S0-1 |
| C20240720 | woman | 45 | Chronic hepatitis B virus | G1 S0-1 |
| C20230922 | woman | 51 | Chronic hepatitis B virus | G1 S1 |
| 202309161 | woman | 51 | Chronic hepatitis B virus | G1 S1 |
| C20240745 | man | 58 | Hepatitis B cirrhosis | G1-2，S3-4 |
| 202501084 | man | 47 | Hepatitis B cirrhosis | G2 S3-4 |
| 202501533 | man | 60 | Hepatitis B cirrhosis | G1，S3-4 |
| C20241065 | woman | 47 | Hepatitis B cirrhosis | G1-2 S3-4 |
| F20231406 | woman | 57 | Hepatitis B cirrhosis | G1-2 S3 |
| 202503507 | woman | 59 | Hepatitis B cirrhosis | G1 S3-4 |
